# Supplementary material for: The hallmarks of a tradeoff in transcriptomes that balances stress and growth functions
Source: mSystems. 2024 Jun 3;9(7):e00305-24. doi: 10.1128/msystems.00305-24 (PMC11264592; doi:10.1128/msystems.00305-24)
Supplement: Supplemental Material — Supplemental text, tables, and figures. [file msystems.00305-24-s0001.docx]

# Supplemental Information

## Other RNAP mutations of special interested from our study

Some mutations predicted to have a specific stress response show no clear existence of one in our study. *RpoC* H419P was commonly found in octanoic acid tolerance studies and thus RNA-sequencing data was gathered for it both on M9 and on octanoic acid. Initially the *rpoC* H419P mutants had trouble growing on octanoic acid until the concentration was lowered, so perhaps an octanoic acid-specific adjustment would have been seen if a higher acid concentration was used. *RpoC* H419P has been previously introduced into *E. coli* and shown to be an adaptation to octanoic acid[^1^](https://paperpile.com/c/NbCbSQ/y1ATZ), so this issue is likely limited to our study.

*RpoB* I966S was commonly selected for in heat tolerance studies[^2–4^](https://paperpile.com/c/NbCbSQ/Vv9k7+jOEDg+9KGBE) and thus we chose to grow it in our study on high temperature conditions. These heat tolerance studies, however, were all carried out using *E. coli B-strains* and there was little effect of this mutation on the RNA-sequencing data so it appears this is a strain specific adaptation.

## Laboratory evolution creates condition-specific convergent mutations

The evolutionary pull towards growth, and thus greed, in ALEs necessitates condition-specific convergent mutations (**Supplemental Table 9**). These mutations are not strictly limited to RNAP. For example, *oxyR* is a common mutation target for evolution in oxidative stress[^5^](https://paperpile.com/c/NbCbSQ/L13aj) and a *topA* mutation was a convergent target in a heat tolerance evolution[^4^](https://paperpile.com/c/NbCbSQ/9KGBE). This trend is widespread among ALEs, as 89% of all evolution experiments in ALEdb contain at least one gene that is mutated in 50% or more of their endpoint strains (calculated by looking at the mutations in all evolved *E. coli* experiments in ALEdb). If excluding RNAP genes, this drops to 80%. While RNAP mutations have the ability to favor growth across a wide variety of conditions, different genes are often better mutational targets for specific conditions.

## The fear vs. greed tradeoff is found in WT and across growth conditions

Fear vs. greed changes are not limited to mutations acquired during evolution, as **Supplemental** **Figure 11** shows how the transcriptome composition falls on the tradeoff line in nutrient limited growth changes. Furthermore, when limiting nutrients drive the culture into the stationary phase, a time series of points shows how the transcriptome composition moves down the f/g tradeoff line. This movement shows how lower growth rates on entry into the stationary phase comes with both a drastic change in transcriptome composition and an increase in stress readiness as has been shown before[^6^](https://paperpile.com/c/NbCbSQ/eGo4y). However, stationary phase cells often have a disconnect between their transcriptomic and proteomic compositions, so the small iModulon movements between the stationary phase samples should be viewed with some skepticism[^6^](https://paperpile.com/c/NbCbSQ/eGo4y). The f/g tradeoff is thus reflected in the various growth states of the WT strain as well as a transition in physiological states.

## Supplemental Tables/Figures

**Supplemental Table 1:** The mutations created for this study and the reason for their inclusion. The column labeled “Midpoint Strains w/ Mutation / Total Strains w/ Mutation and Midpoints” refers to how often these particular mutations are found early in adaptive laboratory evolutions. The fractions listed are the number of corresponding midpoint strains that contain the mutation out of all independent lineages with said mutation. Only strains with both the mutation and midpoint samples available are considered.

| **Gene** | **Mutation** | **Mean Predicted Impact to rpoB-rpoC Interface of Holoenzyme (REU)** | **Mean Predicted Impact to ppGpp Binding (REU)** | **Mutation Frequency (total strains found in)** | **Midpoint Strains w/ Mutation / Total Strains w/ Mutation and Midpoints** | **Reason for Inclusion** |
| --- | --- | --- | --- | --- | --- | --- |
| *rpoA* | G315V (GGC to GTC) | -28.40 | 0.00 | 9 | 4 / 4 | Located nearby to *crp* AR1 binding site, found in *pgi* swap studies |
| *rpoB* | R200P (CGT to CCT) | -60.90 | -9.65 | 6 | 2 / 3 | Found primarily in methionine tolerance experiments |
| *rpoB* | E672K (GAA to AAA) | -28.40 | -22.32 | 23 | 1 / 1 | Common ALEdb mutation, located near the catalytic core of RNAP |
| *rpoB* | G858D (GGT to GAT) | -5.31 | -49.96 | 7 | 0 / 1 | Proximity to *rpoS* binding site |
| *rpoB* | I966S (ATC to AGC) | 0.10 | -3.36 | 18 | 0 / 0 | Found primarily in high temperature experiments |
| *rpoB* | T1037P (ACC to CCC) | -45.91 | -23.69 | 5 | 0 / 0 | Mutation commonly found in drug-resistance experiments |
| *rpoB* | P1100Q (CCG to CAG) | -23.98 | -22.07 | 40 | 7 / 7 | Most common ALEdb mutation, located near the catalytic core of RNAP |
| *rpoB* | G1189C (GGT to TGT) | -5.26 | -9.97 | 19 | 0 / 0 | Common ALEdb mutation, located near the catalytic core of RNAP |
| *rpoC* | N309Y (AAC to TAC) | -216.71 | -91.13 | 2 | 0 / 0 | Found primarily in butanediol tolerance experiments |
| *rpoC* | H419P (CAC to CCC) | -17.15 | -61.99 | 26 | 0 / 0 | Common ALEdb mutation found most often in octanoic tolerance experiments |
| *rpoC* | N720H (AAC to CAC) | -16.65 | 1.02 | 19 | 0 / 0 | Common ALEdb mutation, located near the catalytic core of RNAP |
| *rpoC* | G1055V (GGT to GTT) | -13.91 | -51.71 | 5 | 0 / 0 | Common ALEdb mutation found far from any other mutations included |

**Supplemental Table 2:** Experimental conditions where new RNA-sequencing profiles were generated.

| **Condition** | **Base Media** | **Glucose Concentration** | **Additional Supplements and/or Conditions** |
| --- | --- | --- | --- |
| Glucose | M9 | 4 g/L | none |
| Octanoic | M9 | 4 g/L | 12 mM octanoic acid |
| Methionine | M9 | 4 g/L | 200 mM methionine |
| 42C | M9 | 4 g/L | Run at 42° Celsius |

**Supplemental Table 3:** PDB structures used for structural PyRosetta calculations.

| **RNAP Structural Form** | **PDBs** |
| --- | --- |
| Core Enzyme | 1HQM, 3LU0, 6ASG, 6F6W, 7MKP |
| Holoenzyme | 1L9U, 2A6E, 2CW0, 4MEY, 4YG2, 5BYH, 5NSR, 5NWT, 6EYD |
| Open Complex | 1L9Z, 4G7H, 7D7C, 7KHB |
| Initiation Complex | 4OIO, 4Q4Z, 5IPL, 5IPM, 5IPN, 5VI5, 6VVV |
| Initially Transcribing Complex | 4Q5S |
| Elongation Complex | 2O5I, 2O5J, 6ALF, 6C6T, 6FLQ |
| Pausing Complex | 4GZY, 4GZZ, 6ASX, 6BJS, 6FLP, 6FLQ, 7N4E |
| Backtracked Complex | 4WQS |
| GreA Binding | 4WQT |
| RapA Binding | 4S20, 7M8E, 7MKN, 7MKQ |
| Crp Binding | 6B6H |
| ppGpp Binding | 4JKR |

**Supplemental Table 4**: Possible specific structural impacts of each of the mutations introduced.

| **Gene** | **Mutation** | **Additional Structural Impact Hypotheses** | **Citations** |
| --- | --- | --- | --- |
| rpoA | G315V (GGC to GTC) | RpoA G315 is in the C-terminal domain which is known to interact with promoter DNA and transcription factors, its mutation may affect the specificities of said interactions | [^7^](https://paperpile.com/c/NbCbSQ/nsHRc) |
| rpoB | R200P (CGT to CCT) | In holoenzyme structure, RpoB R200 interacts with sigma region 1.1 which is displaced by promoter DNA in an open complex. In open complex, RpoB R200 directly interacts with non-template strand DNA in the enzyme's main channel. An effect on either of these interactions could affect promoter selectivity. | [^7,8^](https://paperpile.com/c/NbCbSQ/nsHRc+siMJ) |
| rpoB | E672K (GAA to AAA) | RpoB E672 is at the base of the bridge helix, where its mutation likely affects promoter DNA-RNAP interactions, possibly accounting for its effect on gene selectivity. | [^9^](https://paperpile.com/c/NbCbSQ/npCyW) |
| rpoB | G858D (GGT to GAT) | RpoB G858 is relatively near the Flap-Tip subdomain, a domain of the beta subunit that interacts with sigma region 4 and therefore may impact sigma recognition of the -35 region. | [^10^](https://paperpile.com/c/NbCbSQ/t0Ha8) |
| rpoB | I966S (ATC to AGC) | Unclear - has only a minor predicted structural impact on ppGpp binding or the rpoB-rpoC interface and not near (< 20 angstroms) any known structural region of interest. The mutation’s prevalence in high temperature conditions in *E. coli B-strain* ALEs infers it likely stabilizes RNAP in said strains. | [^8^](https://paperpile.com/c/NbCbSQ/siMJ) |
| rpoB | T1037P (ACC to CCC) | In the beta prime subunit, rpoB G1037 is in sequence insertion 3 (SI3). The function of SI3 is unclear, but SI3 is contiguous with the trigger loop and thus the mutation may affect catalysis. | [^11^](https://paperpile.com/c/NbCbSQ/7dEke) |
| rpoB | P1100Q (CCG to CAG) | The rpoB P1100Q substitution is near the base of a helix in the beta prime subunit that interacts with ppGpp binding site 1, which could potentially impact promoter selectivity. | [^12^](https://paperpile.com/c/NbCbSQ/765pJ) |
| rpoB | G1189C (GGT to TGT) | RpoB G1189 is near the base of the bridge helix, where its mutation likely affects promoter DNA-RNAP interactions, possibly accounting for its effect on gene selectivity. | [^8,11^](https://paperpile.com/c/NbCbSQ/7dEke+siMJ) |
| rpoC | N309Y (AAC to TAC) | Beta prime N309Y is close to the interface of beta prime with sigma regions 1.1 and 1.2, parts of the enzyme that directly affect promoter specificity. | [^8^](https://paperpile.com/c/NbCbSQ/siMJ) |
| rpoC | H419P (CAC to CCC) | Beta prime H419P has been identified previously (Chen et al 2020. Metab Eng. 61:120-130. PMID: 32474056) where it was argued that this substitution might affect gene expression by virtue of its proximity to ppGpp binding site 1. | [^1^](https://paperpile.com/c/NbCbSQ/y1ATZ) |
| rpoC | N720H (AAC to CAC) | RpoC N720 is near the base of the bridge helix, where its mutation likely affects promoter DNA-RNAP interactions, possibly accounting for its effect on gene selectivity. | [^8^](https://paperpile.com/c/NbCbSQ/siMJ) |
| rpoC | G1055V (GGT to GTT) | This residue is near no known region of interest and is found in a few unrelated evolutions. Despite this, the mutation impacts the transcriptome similarly to the other mutations. The large size shift from glycine to valine possibly has a large structural impact on regions distant from the mutation. |  |

**Supplemental Table 5:** Sigma factors, their regulated genes, and the average expression change among the mutated strains created for this study.

| **Sigma Factor** | **Genes Regulated (according to RegulonDB**[^13^](https://paperpile.com/c/NbCbSQ/YA7HU)**)** | **Average Expression Change (log_2_ tpm)** |
| --- | --- | --- |
| Sigma 24 (*rpoE)* | *ahpF, apaG, apaH, bacA, bamA, bamB, bamC, bamD, bamE, bepA, bhsA, cca, clpX, degP, der, dnaE, dsbC, eptB, essD, fabZ, fadM, fkpA, ftnB, fusA, greA, gspA, gspB, hcp, hcr, hpf, ibaG, insK, lgoR, lhr, lon, lptA, lptB, lptD, lpxA, lpxB, lpxD, lpxP, lyxK, malQ, mscM, mzrA, narV, narW, opgG, opgH, pdxA, phoQ, plsB, prfB, psd, ptsN, rapZ, rclA, rclC, recJ, recR, rfaD, rnhB, rnlB, rpoD, rpoE, rpoH, rpoN, rrrD, rseA, rseB, rseC, rseD, rseP, rsmA, rutR, rzpD, sbmA, sgbE, sgbH, sgbU, sixA, skp, surA, tufA, uspD, waaC, waaF, waaL, wza, wzb, wzc, yaiW, ybaB, ybfG, ydhI, ydhJ, ydhK, yeaY, yfeK, yfeS, yfeX, yfeY, yfgD, yggN, yghF, ygiM, yhjJ, yiaK, yiaL, yiaM, yiaN, yiaO, yicI, yicJ, yidQ, yieE, yieF, yiiS, yoaC, yqjA, ytfJ* | -0.041 |
| Sigma 28 (*rpoF*) | *aer, cheA, cheB, cheR, cheW, cheY, cheZ, flgK, flgL, flgM, flgN, fliA, fliC, fliD, fliE, fliF, fliG, fliH, fliI, fliJ, fliK, fliL, fliM, fliN, fliO, fliP, fliQ, fliR, fliS, fliT, fliZ, flxA, hemL, ispE, lolB, modA, modB, modC, motA, motB, oppA, oppB, oppC, oppD, oppF, pdeH, ppdA, ppdB, ppdC, prs, recC, tap, tar, tcyJ, trg, tsr, ves, yafW, yafX, ycgR, yecF, ygbK, ygdB, yhiL, yjcS, ykfB, ykfF, ykfG, ykfH, ykfI, ynjH* | -0.053 |
| Sigma 32 (*rpoH*) | *ackA, adiC, alaA, bssS, can, cas1, cas2, casD, casE, clpB, clpP, clpX, cnoX, cra, creA, creB, creC, crr, dnaJ, dnaK, dsbC, fkpB, ftsH, fxsA, gapA, glnS, groL, groS, grpE, hflC, hflK, hflX, hfq, holC, hslO, hslR, hslU, hslV, hspQ, htpG, htpX, ibpA, ibpB, ileS, ispH, lapA, lapB, ldhA, lipB, lnt, lon, lspA, mbiA, metA, mhpT, miaA, mlc, mngA, mngB, mpaA, mutL, mutM, narP, nfuA, nusB, osmF, pgpA, pgpC, phoP, phoQ, pncC, pphA, ppiD, prlC, ptsH, ptsI, pyrF, raiA, rapA, rdgB, recJ, rfaD, ribE, rlmE, rnlA, rpmE, rpoD, rsmJ, sdaA, slt, tadA, thiL, topA, trmA, tyrR, valS, waaC, waaF, waaL, xerD, yafD, yafE, yafU, ybeD, ybeX, ybeY, ybeZ, ybfE, yccE, yceI, yceJ, yciH, ycjF, ycjX, ycjY, ydeO, ydhQ, yeaD, yehR, yehW, yehX, yehY, yfbR, yfjV, yhdN, yiaA, yibA, yjaZ, yjhG, yjhH, yjhI, yjiT, ymjC, yrdA, yrfG, zntR* | -0.040 |
| Sigma 38 (*rpoS*) | *aceE, aceF, acnA, acs, actP, ada, adhE, aidB, aldB, alkA, alkB, ansP, appA, appB, appC, appY, araF, araG, araH, ariR, artI, artM, artP, artQ, asr, astA, astB, astC, astD, astE, baeR, baeS, blc, blr, bolA, bsmA, btsT, btuF, cbpA, cbpM, cfa, cpxA, cpxR, crr, csgA, csgB, csgC, csgD, csgE, csgF, csgG, csiE, csrA, ddpA, ddpB, ddpC, ddpD, ddpF, ddpX, dgcC, dhaK, dhaL, dhaM, dhaR, dinB, dkgB, dmsD, dnaN, dps, ecnB, elaB, eno, envC, epd, evgA, evgS, fadL, fau, fbaA, fbaB, fic, folK, frdA, frdB, frdC, frdD, ftsA, ftsQ, ftsZ, fumC, gabD, gabP, gabT, gadA, gadB, gadC, gadE, gadW, gadX, galE, galK, galM, galT, gapA, glgA, glgC, glgP, glgS, glk, glsA, gltB, gltD, gltF, gor, gpmA, gpmM, gyrB, hchA, hdeA, hdeB, hdeD, hdfR, hmp, hofM, hofN, hofO, htrE, hyaA, hyaB, hyaC, hyaD, hyaE, hyaF, ihfA, ihfB, ilvY, iraD, katE, kbp, ldcC, lpd, lsrA, lsrB, lsrC, lsrD, lsrF, lsrG, luxS, mdtA, mdtB, mdtC, mdtD, mdtE, mdtF, metK, mglA, mglB, mglC, mlrA, mnmG, mpl, msyB, mtn, murP, murQ, murR, mutS, narU, nhaA, nhaR, ompF, ompN, osmB, osmC, osmE, osmF, osmY, otsA, otsB, oxyR, pabA, patA, patD, pcnB, pdeC, pdeR, pdhR, pfkA, pfkB, pgi, pgk, phoU, phr, poxB, pphA, ppk, ppx, pqiA, pqiB, pqiC, proP, proV, proW, proX, pstA, pstB, pstC, pstS, puuA, puuB, puuC, puuD, puuE, puuP, puuR, pykA, pykF, ravA, recF, rhaR, rhaS, rpoE, rpoH, rraA, rsd, rseA, rseB, rseC, rseD, rsmG, rssA, rssB, setA, sgrT, sodC, sohB, speB, speC, speG, sra, sucA, sucB, sucC, sucD, talA, tam, tktB, tolC, topA, tpiA, treA, treF, uspB, viaA, wrbA, xapA, xapB, xthA, yabI, yadS, yadV, yafN, yafO, yafP, ybaT, ybgA, ybiI, ybjP, yccJ, ycgZ, yciE, yciF, yciG, yciT, yciZ, ydbD, ydbK, ydcS, ydcT, ydcU, ydcV, yddG, ydhT, ydhU, ydhV, ydhW, ydhX, ydhY, yehW, yehX, yehY, yeiL, yffO, yffP, yggE, ygiB, ygiC, yhfG, yhiD, yhiM, yhjG, yiaG, yibQ, yihG, yjcH, ymgA, ymgC, ymjE, ynfB, ynfE, ynfF, ynfG, ynfH, ytfK, ytiC, ytiD, znuA* | -0.332 |
| Sigma 54 (*rpoN*) | *acrD, actP, amtB, argT, aslB, astA, astB, astC, astD, astE, atoA, atoB, atoD, atoE, chaC, dcuD, ddpA, ddpB, ddpC, ddpD, ddpF, ddpX, emrD, fdhF, fhlA, flhC, flhD, focB, glnA, glnG, glnH, glnK, glnL, glnP, glnQ, glpQ, gltI, gltJ, gltK, gltL, gnsA, hisJ, hisM, hisP, hisQ, htpG, hycA, hycB, hycC, hycD, hycE, hycF, hycG, hycH, hycI, hydN, hyfA, hyfB, hyfC, hyfD, hyfE, hyfF, hyfG, hyfH, hyfI, hyfJ, hyfR, hypA, hypB, hypC, hypD, hypE, hypF, ibpB, nac, nikA, nikB, nikC, nikD, nikE, nikR, norV, norW, patA, potF, potG, potH, potI, prpB, prpC, prpD, prpE, pspA, pspB, pspC, pspD, pspE, pspG, puuP, radD, relA, rhaD, rpoE, rpoH, rseA, rseB, rseC, rseD, rtcA, rtcB, rtcR, rutA, rutB, rutC, rutD, rutE, rutF, rutG, ssnA, xapB, xdhA, xdhB, xdhC, yaaU, yahE, yeaG, yeaH, ygfK, yhdW, yhdX, yhdY, yhdZ, ymjE, zraP, zraR, zraS* | -0.057 |
| Sigma 70 (*rpoD*) | *accA, accB, accC, accD, aceA, aceB, aceE, aceF, aceK, acnA, acnB, acpP, acpS, acrA, acrB, acrD, acrE, acrF, acrR, acrZ, acs, actP, ada, add, adeD, adhE, adiA, agaA, agaC, agaD, agaI, agaR, agaS, agaV, agaW, ahpC, ahpF, aidB, alaC, alaE, aldA, alkA, alkB, allB, allC, allD, allE, alsA, alsB, alsC, alsE, alsR, amiA, amiB, ampC, ampG, amyA, ansB, appA, appB, appC, appY, apt, araA, araB, araC, araD, araE, araF, araG, araH, araJ, arcA, arfA, argA, argB, argC, argD, argE, argF, argG, argH, argI, argO, argR, argS, ariR, aroA, aroB, aroD, aroF, aroG, aroH, aroK, aroL, aroM, aroP, arsB, arsC, arsR, artI, artJ, artM, artP, artQ, ascB, ascF, asnA, asnC, aspA, asr, astA, astB, astC, astD, astE, atpA, atpB, atpC, atpD, atpE, atpF, atpG, atpH, atpI, azoR, bamC, bcp, bcsB, bcsE, bcsF, bcsG, bcsZ, bdcA, betA, betB, betI, betT, bglJ, bglX, bhsA, bioA, bioB, bioC, bioD, bioF, bluF, bluR, bolA, bssS, btuB, btuD, btuE, btuF, cadA, cadB, cadC, caiA, caiB, caiC, caiD, caiE, caiF, caiT, can, carA, carB, cas1, cas2, cas3, casA, casB, casC, casD, casE, cbl, cbpA, cbpM, ccmA, ccmB, ccmC, ccmD, ccmE, ccmF, ccmG, ccmH, ccp, cdd, cecR, cedA, cfa, chiA, cho, chpB, chpS, cirA, clpA, clpB, clpP, clpX, cmk, cmoM, coaD, cobS, cobT, cobU, codA, codB, copA, corA, cpdB, cpxA, cpxP, cpxR, creA, creB, creC, creD, crp, crr, csgA, csgB, csgC, csgD, csgE, csgF, csgG, csiE, cspA, cspB, cspC, cspD, cspE, csrA, cstA, cusA, cusB, cusC, cusF, cusR, cusS, cutA, cvpA, cyaA, cydA, cydB, cydC, cydD, cynR, cynS, cynT, cynX, cyoA, cyoB, cyoC, cyoD, cyoE, cysA, cysB, cysC, cysD, cysG, cysH, cysI, cysJ, cysK, cysM, cysN, cysP, cysU, cysW, cytR, dacC, dadA, dadX, dam, damX, dapA, dapB, dapD, dapE, dapF, dctA, dctR, dcuA, dcuB, dcuD, dcuR, dcuS, ddlB, deaD, decR, def, degQ, degS, deoA, deoB, deoC, deoD, dgcN, dgcT, dgcZ, dgoA, dgoD, dgoK, dgoR, dgoT, dinB, dinF, dinG, dinI, dinJ, dkgA, dksA, dmsA, dmsB, dmsC, dmsD, dnaA, dnaG, dnaN, dppA, dppB, dppC, dppD, dppF, dps, dsbA, dsbC, dsdA, dsdC, dsdX, dtpA, dusB, dut, dxs, ebgA, ebgC, ecpA, eda, edd, efeU, efp, emrA, emrB, eno, entA, entB, entC, entD, entE, entF, entH, entS, envZ, epd, eptB, era, evgA, evgS, exbB, exbD, exuR, fabA, fabB, fabD, fabF, fabG, fabH, fadA, fadB, fadD, fadI, fadJ, fadR, fau, fbaA, fdnG, fdnH, fdnI, fdx, feaB, feaR, fecI, fecR, feoA, feoB, feoC, fepA, fepB, fepC, fepD, fepE, fepG, fes, fhuA, fhuB, fhuC, fhuD, fhuF, fimA, fimB, fimC, fimD, fimE, fimF, fimG, fimH, fimI, fis, fiu, fixA, fixB, fixC, fkpB, fldB, flgA, flgB, flgC, flgD, flgE, flgF, flgG, flgH, flgI, flgJ, flgM, flgN, flhA, flhB, flhC, flhD, flhE, fliA, fliD, fliE, fliF, fliG, fliH, fliI, fliJ, fliK, fliL, fliM, fliN, fliO, fliP, fliQ, fliR, fliS, fliT, fliZ, flu, fmt, fnr, focA, folA, folE, folK, fpr, frc, frdA, frdB, frdC, frdD, frlA, frlB, frlC, frlD, frlR, frr, frsA, fruA, fruB, fruK, ftnA, ftnB, ftsA, ftsH, ftsI, ftsK, ftsL, ftsQ, ftsW, ftsZ, fucA, fucI, fucK, fucO, fucP, fucR, fucU, fumA, fumB, fumC, fur, gabD, gabP, gabT, gadA, gadB, gadC, gadE, gadX, galE, galK, galM, galP, galR, galS, galT, gapA, garK, garL, garP, garR, gatA, gatB, gatC, gatD, gatY, gatZ, gcd, gcl, gcvA, gcvH, gcvP, gcvR, gcvT, gdhA, gfcA, glcA, glcB, glcC, glcD, glcE, glcF, glcG, glgA, glgB, glgC, glgP, glgX, glk, glmS, glmU, glnA, glnB, glnG, glnL, glnQ, glnS, gloA, glpA, glpB, glpC, glpD, glpF, glpK, glpQ, glpT, glpX, glrK, gltA, gltB, gltD, gltF, gltI, gltJ, gltK, gltL, gltX, gluQ, glxK, glxR, glyA, glyQ, glyS, gmk, gnd, gnsA, gntK, gntP, gntR, gntT, gntU, gor, gph, gpmA, gpsA, gpt, grcA, greA, groL, groS, grxB, grxC, grxD, gsk, gspA, gspB, gspC, gspD, gspE, gspF, gspG, gspH, gspJ, gspK, gspL, gspM, gspO, guaA, guaB, gutM, gutQ, gyrA, gyrB, hchA, hdeA, hdeB, hdeD, hemA, hemB, hemF, hemH, hemN, hflC, hflD, hflK, hflX, hfq, hha, hicA, hicB, higA, higB, hipA, hipB, hisA, hisB, hisF, hisH, hisI, hisJ, hisM, hisP, hisQ, hisS, hns, hofB, hofC, hokD, hpf, hpt, hscA, hscB, hslJ, htrE, hupB, hyaA, hyaB, hyaC, hyaD, hyaE, hyaF, hybA, hybB, hybC, hybD, hybE, hybF, hybG, hybO, hyi, hypF, icd, idnK, idnR, ihfB, ileS, ilvA, ilvB, ilvC, ilvD, ilvE, ilvG, ilvH, ilvI, ilvM, ilvN, ilvY, inaA, infA, infB, infC, intQ, intS, iraD, iraM, iraP, iscA, iscR, iscS, iscU, iscX, ispA, ispH, katG, kbaY, kbaZ, kbl, kdpA, kdpB, kdpC, kdsA, kdsB, kdsC, kdsD, lacA, lacI, lacY, lacZ, lamB, lapA, lapB, ldtC, leuA, leuB, leuC, leuD, leuO, lexA, lgt, lipB, livF, livG, livH, livJ, livK, livM, lldD, lldP, lldR, loiP, lolA, lon, lpd, lptA, lptB, lptC, lptD, lpxC, lpxT, lrp, lspA, lsrK, lsrR, lysA, lysC, lysO, lysP, lysR, lysU, lyxK, malE, malF, malG, malI, malK, malM, malP, malQ, malS, malT, malX, malY, manA, manX, manY, manZ, marA, marB, marR, mazE, mazF, mcbA, mdh, mdtE, mdtF, mdtI, mdtJ, melA, melB, melR, menA, mepH, metA, metB, metC, metF, metG, metH, metI, metJ, metK, metL, metN, metQ, mfd, mglA, mglB, mglC, mgtA, mhpA, mhpB, mhpC, mhpD, mhpE, mhpF, mhpR, miaA, mioC, mlc, mlrA, mltF, mngB, mngR, mnmG, mntH, mntP, mntS, modA, modB, modC, moeA, moeB, mpl, mprA, mqsA, mqsR, mraY, mraZ, mreB, mreC, mreD, mrp, msrB, mtlA, mtlD, mtlR, mtn, mtr, mukB, mukE, mukF, murC, murD, murE, murF, murG, murI, murP, murQ, murR, mutL, mzrA, nadB, nadE, nagA, nagB, nagC, nagE, nanA, nanE, nanK, nanT, napA, napB, napC, napD, napF, napG, napH, narG, narH, narI, narJ, narK, narL, narU, narX, ndh, nei, nemA, nemR, nfeF, nfo, nfsA, nfsB, nfuA, nhaA, nhaR, nirB, nirC, nirD, nlpD, nnr, nohA, npr, nrdA, nrdB, nrdD, nrdE, nrdF, nrdG, nrdH, nrdI, nrdR, nrfA, nrfB, nrfC, nrfD, nrfE, nrfF, nrfG, nudB, nuoA, nuoB, nuoC, nuoE, nuoF, nuoG, nuoH, nuoI, nuoJ, nuoK, nuoL, nuoM, nuoN, nupC, nupG, nusA, nusB, obgE, ompA, ompC, ompF, ompN, ompR, ompX, opgG, opgH, osmB, osmC, osmE, osmY, oxc, oxyR, paaA, paaB, paaC, paaD, paaE, paaF, paaG, paaH, paaI, paaJ, paaK, paaX, paaY, pabA, panB, panC, panD, panF, patZ, pck, pcm, pcnB, pdeL, pdhR, pdxJ, pepA, pepD, pepP, pfkA, pflA, pflB, pgi, pgk, pgpA, pgpB, pheA, pheP, phnC, phnD, phnF, phnG, phnH, phnI, phnJ, phnK, phnL, phnM, phnN, phnO, phnP, phoA, phoB, phoE, phoH, phoR, phoU, pitB, pldA, plsX, pncB, pnp, pntA, pntB, polB, poxB, ppdD, ppiA, ppiD, ppsA, pqiA, pqiB, pqiC, preA, preT, prfA, prfC, priB, prlF, prmA, prmC, proP, proS, proV, proW, proX, prpR, prs, psiE, psiF, pspF, pssA, pstA, pstB, pstC, pstS, pta, pth, ptrA, ptsG, ptsH, ptsI, ptsN, purA, purB, purC, purD, purE, purF, purH, purK, purL, purM, purN, purR, purT, putA, putP, pykA, pykF, pyrC, pyrD, pyrF, pyrH, qseB, qseC, rapA, rapZ, rarA, rbfA, rbn, rbsA, rbsB, rbsC, rbsD, rbsK, rbsR, rcnA, rcnB, rcnR, rcsA, rcsB, rcsD, recA, recB, recC, recD, recF, recG, recJ, recN, recO, recQ, recX, relA, relB, relE, rep, rfaD, rfe, rffC, rffG, rffH, rffM, rhaA, rhaB, rhaD, rhaR, rhaS, rhaT, ribA, ribD, ribE, ribF, rihB, rimK, rimM, rimP, rlmE, rluA, rnb, rnc, rnd, rne, rnlA, rnlB, rpe, rpiB, rplB, rplC, rplD, rplI, rplP, rplQ, rplS, rplT, rplU, rplV, rplW, rpmA, rpmC, rpmI, rpoA, rpoD, rpoE, rpoH, rpoN, rpoS, rpoZ, rpsA, rpsB, rpsC, rpsD, rpsF, rpsJ, rpsK, rpsM, rpsO, rpsP, rpsQ, rpsR, rpsS, rpsU, rraA, rraB, rsd, rseA, rseB, rseC, rseD, rsmG, rsmH, rutR, ruvC, safA, sbcC, sbcD, sdhA, sdhB, sdhC, sdhD, sdiA, secB, secG, serA, serC, setA, sfsA, sgbE, sgbH, sgbU, sgrR, sgrT, shiA, sixA, slmA, slp, sodA, sodB, sohB, soxR, soxS, speA, speB, speD, speE, spoT, spy, srkA, srlA, srlB, srlD, srlE, srlR, ssb, ssnA, ssuA, ssuB, ssuC, ssuD, ssuE, stpA, sucA, sucB, sucC, sucD, sufA, sufB, sufC, sufD, sufE, sufS, sulA, sutR, sxy, symE, tatA, tatB, tatC, tatD, tcyJ, tdh, tfaQ, thiB, thiL, thiP, thiQ, thrA, thrB, thrC, thyA, tnaA, tnaB, tomB, tonB, topA, torA, torC, torD, torR, tpiA, tpx, treB, treC, treR, trmD, trmH, trpA, trpB, trpC, trpD, trpE, trpR, trpS, truB, trxA, trxC, tsaE, tsf, tsr, tsx, ttdA, ttdB, ttdT, tufA, tufB, tynA, tyrA, tyrB, tyrP, tyrR, ubiA, ubiC, ubiG, ubiH, ubiI, ubiX, udp, ugpA, ugpB, ugpC, ugpE, ugpQ, uhpT, uidR, ulaA, ulaB, ulaC, ulaD, ulaE, ulaF, umpG, umpH, umuC, umuD, upp, uraA, uspA, uvrA, uvrB, uvrC, uvrD, uvrY, uxaA, uxaB, uxaC, uxuA, uxuB, uxuR, valS, waaA, waaB, waaC, waaF, waaG, waaH, waaJ, waaL, waaO, waaP, waaQ, waaS, waaU, waaY, waaZ, wcaA, wcaB, wecB, wecC, wecE, wza, wzb, wzc, wzxE, wzyE, wzzE, xapA, xapB, xapR, xerC, xerD, xseA, xseB, xylE, yacC, yadS, yadV, yafN, yafO, yafP, yafQ, yagK, yaiA, yajG, yajO, ybbW, ybbY, ybdN, ybdZ, ybeD, ybhF, ybhG, ybhL, ybhR, ybhS, ybiB, ybjC, ybjN, ycaR, yccA, ycdZ, yceI, yceJ, ycgZ, ychA, ychF, ychH, ychO, ychQ, yciB, yciC, yciE, yciH, yciW, yciX, ydeI, ydeJ, ydeM, ydeN, ydeO, ydeP, ydfA, ydfC, ydfE, ydfW, ydfX, ydjM, yeaE, yeaR, yebB, yebC, yebE, yebG, yefM, yegR, yegZ, yehF, yeiB, yfaE, yfdE, yfdV, yfdX, yffB, yfgG, yfiB, yfiR, ygeA, ygeH, ygfB, yhaV, yhbE, yhdT, yhfA, yhgH, yhhY, yhiD, yhjR, yhjX, yiaK, yiaL, yiaM, yiaN, yiaO, yibN, yifL, yigA, yigB, yihI, yihS, yihT, yihU, yihV, yjbE, yjbF, yjbG, yjbH, yjcH, yjjQ, yjjZ, ykgM, ykgO, ykgR, ymgA, ymgC, ymiA, ynfE, ynfF, ynfG, ynfH, yoaE, yoaG, yobF, yoeB, yojI, ypfN, yqhD, yqjA, yrbG, ysgA, ytiC, ytiD, zinT, zntA, znuB, znuC, zwf* | -0.060 |

**Supplemental Table 6:** The top five principal component analysis components of the **A** matrix of PRECISE 2.0 and their contributing factors.

| **PC Rank** | **Variance Explained** | **Top 5 Contributing iModulons (percentage of total weight)** |
| --- | --- | --- |
| 1 | 17.6% | RpoS (5.72%), FlhDC-2 (4.15%), FliA (3.10%), GadX (2.59%), Translation (1.91%) |
| 2 | 14.2% | ppGpp (3.55%), RpoS (2.75%), Fnr-2 (2.64%), PurR (2.53%), Fnr-1 (2.38%) |
| 3 | 8.2% | RpoS (2.06%), FlhDC-2 (1.97%), DksA-related (1.84%), Crp-related (1.82%), Crp-1 (1.80%) |
| 4 | 6.1% | Anaero-related (2.57%), Fnr-2 (2.53%), Lrp (2.41%), Fnr-1 (2.17%), ArcA (2.15%) |
| 5 | 5.5% | FlhDC-2 (5.31%), FliA (4.93%), RpoS (4.52%), GadX (4.02%), RhaS (3.31%) |

**Supplemental Table 7:** Genes in various fear vs. greed tradeoff associated iModulons, ordered by iModulon weightings from highest to lowest.

| **iModulon** | **Genes** | **iModulonDB Link** |
| --- | --- | --- |
| Translation | *rplV, rpsS, rpsC, rplW, rplB, rplP, rplD, rpmC, rpsQ, rplC, rpmD, rpsJ, rplR, rpsE, rplO, rpsR, rplF, rplI, rplA, priB, rpsF, secY, rpsH, rplK, rpsN, fusA, rplM, rplE, rpsG, rpsI, rpsD, rplQ, rpoA, tsf, trmD, rimM, rbfA, rplX, rplS, rpmJ, rpsL, rpsK, truB, rplY, rplN, rpsP, rplL, rpsB, infB, aceF* | https://imodulondb.org/iModulon.html?organism=e_coli&dataset=precise2&k=79 |
| ppGpp | *plaP, suhB, rimO, dusB, ydhC, potA, rlmG, yidD, gpt, rluB, queA, fis, rnpA, ydiY, yfhL, ydfO, yegQ, potB, rhlE, ygiQ, rph, rlmC, yegD, rlmF, yciH, pyrF, queD, dusC, pyrD, rpsT, rpsU, mntP, tusA, tsaB, pcnB, ndk, rluC, opgC, yceA, epmA, dbpA, rlmN, yhhQ, fadL, thiI, upp, mltD, mnmA, srmB, ydjX, mnmG, gsk, folK, ycaO, mrcA, rlhA, infA, cmk, cspF, dgcJ, rimI, rplU, miaB, murJ, lysO, trmA, apt, ortT, yggI, rsxB, trmL, recQ, yhbE* | https://imodulondb.org/iModulon.html?organism=e_coli&dataset=precise2&k=177 |
| RpoS | *yiaG, ygaM, ycaC, yahO, blc, katE, talA, ycgB, poxB, ybhP, elaB, osmY, otsB, tktB, yodD, ytjA, yciG, yegP, ecnB, patA, yeaG, osmF, ahr, yebV, yjdN, wrbA, yfcG, yccJ, yeaH, ynaL, clsB, yghA, otsA, ggt, msyB, adhP, ybgS, ymgE, yhcO, yeaQ, psiF, yhbO, kbp, yegS, bfr, ybgA, ybdK, fbaB, yehY, ydhS, ybaY, yahK, dgcM, sra, yehW, gabD, yphA, gabT, amyA, yehX, osmC, yohF, yedP, fic, ygdI, dps, yqjE, yqjD, yebF, osmE, yhjG, ydiZ, ybhN, yqjK, yqjC, yjdJ, ydeI, treA, mlrA, aldB, lhgO, tam, yhjY, mcbA, ygiW, yfdC, ybaA, yhfG, curA, gabP, ldtE, sodC, yehE, yciF, yqjG, yjdI, yfiL, ysgA, ydcS, mcbR, yncG, ybjP, yhdW, yohC, ydcK, csiD, ycaP, hchA, chaB, ybeL, yniA, yjgH, yajO, pdeR, yghX, ybiI, yliI, rclA, ldcC, ybhB, ybiO, dkgA, aidB, yjfJ, yjfI* | https://imodulondb.org/iModulon.html?organism=e_coli&dataset=precise2&k=188 |
| GadX | *hdeB, hdeD, hdeA, gadA, gadB, gadE, yhiD, gadC, yhiM, rcsB, slp, mdtE, dctR, aidB, mdtF, glsA, ybaT* | https://imodulondb.org/iModulon.html?organism=e_coli&dataset=precise2&k=99 |

**Supplemental Table 8:** ALEdb experiments used in this paper and their respective ALEdb and PRECISEdb names, if available.

| **Name on Plots** | **Short Explanation** | **Publication** | **ALEdb Link** | **PRECISEdb 2.0 Name** |
| --- | --- | --- | --- | --- |
| 2CS KOs | Two component systems were KO’d and evolved | n/a | n/a | Two Component Systems |
| 42C | Heat tolerance evolution | [^14^](https://paperpile.com/c/NbCbSQ/1n3vR) | https://aledb.ucsd.edu/ale/project/1/ | 42C Evolution |
| Acid | Acid tolerance evolution | [^15^](https://paperpile.com/c/NbCbSQ/wH4VL) | n/a | Acid |
| AdnB KO | *AdnB* was KO’d | n/a | n/a | adnB |
| AntibioticICA | Antibiotic tolerance | n/a | n/a | AntibioticICA |
| Control | M9 glucose growth with no stressors | [^16^](https://paperpile.com/c/NbCbSQ/AYnWA) | n/a | Control |
| Cra/Crp KOs | Cra/Crp KO’d | [^16^](https://paperpile.com/c/NbCbSQ/AYnWA) | n/a | Cra/Crp |
| Crp ARs | KO’d different binding regions of crp | [^16^](https://paperpile.com/c/NbCbSQ/AYnWA) | n/a | Crp ARs |
| Enzyme Promiscuity | Evolved to enable growth on new substrates | [^17^](https://paperpile.com/c/NbCbSQ/EUxlm) | n/a | Enzyme Promiscuity |
| Falsely Predicted Essential KOs | KO’d genes that were false positively predicted to be essential | [^18^](https://paperpile.com/c/NbCbSQ/u9XL1) | n/a | False Positives |
| Fur KOs | *Fur* KO’d | [^19^](https://paperpile.com/c/NbCbSQ/fUo7u) | n/a | Fur |
| Glucose Evolution | Evolved on M9 glucose with no stressors | [^20^](https://paperpile.com/c/NbCbSQ/APL8R) | https://aledb.ucsd.edu/ale/project/15/ | Glucose Evolution |
| HOT ALE | Heat tolerance evolution | n/a | n/a | HOT ALE |
| Misc |  | n/a | n/a | Misc |
| Nac/NtrC KOs | Evolved *Nac*/*NtrC* KO’s | dissertation - <https://escholarship.org/uc/item/0k4709x6> | n/a | Nac/NtrC |
| Naphthoquinone | Evolved obligative naphthoquinone users | [^21^](https://paperpile.com/c/NbCbSQ/Utkna) | n/a | Naphthoquinone |
| OmpR KOs | *OmpR* was KO’d and grown under osmotic stress | [^22^](https://paperpile.com/c/NbCbSQ/uNVqY) | n/a | OmpR |
| Oxidative | Oxidative stress conditions | [^23^](https://paperpile.com/c/NbCbSQ/WUg5P) | n/a | Oxidative |
| OxyR KO ALE | *OxyR* KO’d and evolved | [^5^](https://paperpile.com/c/NbCbSQ/L13aj) | n/a | OxyR ALE |
| RpoB Knock-in | Two *rpoB* SNP mutations introduced | [^24^](https://paperpile.com/c/NbCbSQ/f5alj) | n/a | RpoB Knock-in |
| pdhR ALE | *pdhR* was KO’d and evolved | n/a | n/a | pdhR-ALE |
| pH ALE | Evolved at various pH’s | n/a | n/a | pH ALE |
| Pseudogene Repair | KO’d *entC*, *menF*, and *ubiC* to induce iron import stress and evolved | [^25^](https://paperpile.com/c/NbCbSQ/1l8TR) | n/a | Pseudogene Repair |
| Respiratory Quinone | *MenF* and *ubiC* deletions and evolutions on glucose | n/a | n/a | Respiratory Quinone |
| ROS TALE | Evolved cells on various concentrations of paraquat | [^26^](https://paperpile.com/c/NbCbSQ/olcim) | https://aledb.ucsd.edu/ale/project/184/ | ROS TALE |
| Substrate - switching Evolution | Evolved on alternating carbon sources | [^27^](https://paperpile.com/c/NbCbSQ/PYwrx) | https://aledb.ucsd.edu/ale/project/42/ | Substrate-switching Evolution |
| SvNS PGI | Various import metabolic genes were KO’d and replaced with exogenous copies, then evolved | Unpublished extension of Sanberg et al. 2020[^28^](https://paperpile.com/c/NbCbSQ/BXnu8) | https://aledb.ucsd.edu/ale/project/51/ | SvNS PGI |
| Various Carbon | Grown on a variety of carbon sources | n/a | n/a | ICA |
| yTF KO | Unknown function TF’s were KO’d | [^29^](https://paperpile.com/c/NbCbSQ/N4c6u) | n/a | yTF |

**Supplemental Table 9:** ALE experiments with likely synergistic mutations. All genes mutated in at least half of each experiments’ samples are shown.

| **ALE Study** | **RNAP Genes Mutated** | **Other Genes Mutated** |
| --- | --- | --- |
| 42C | *rpoC* (5/10) | mprA (5/10) |
| Substrate-Switching | *rpoC* (7/9) | *glpK* (5/9), *ptsP* (5/9) |
| SvNS PGI | *rpoA* (20/33) | *arcB* (29), *gatC* (29), *glpR* (29), *bgIJ* (25), *gabP* (25), *narU* (25), *wbbH* (25), *ykfH* (25), *yphG* (25), *oxyR* (21) all out of 33 |
| Glucose Evolution | *rpoB* (7/8) |  |
| ROS TALE | *rpoB* (17/17) | *aceE* (16), *ygfZ* (16), *iscR* (15), *glnX* (9) all out of 17 |


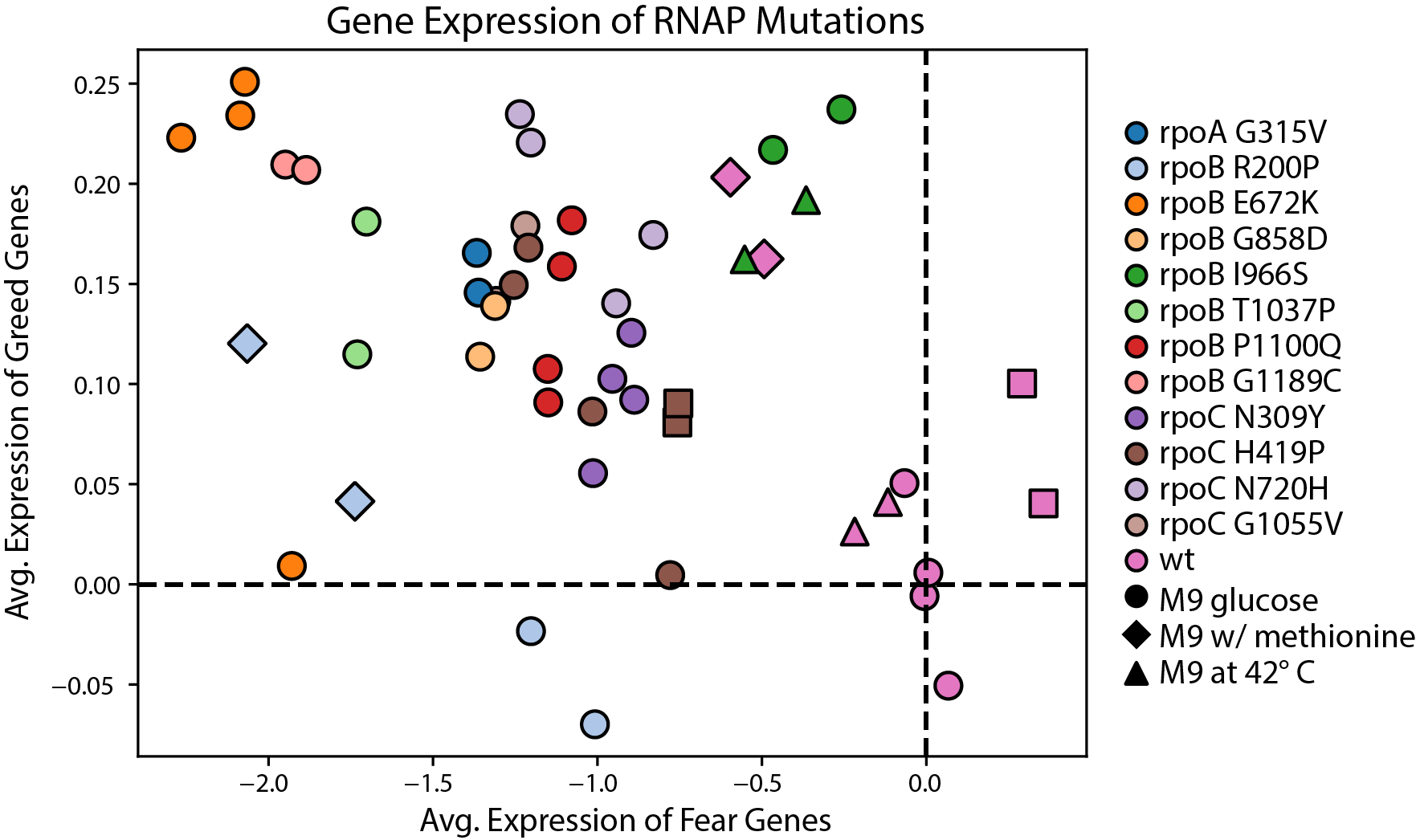


**Supplemental Figure 1 -** The average expression values of all replicates from this study are shown for “fear” and “greed” genes. Gene expression is in log TPM and centered on the “wt M9 glucose” condition. “Fear” genes are those that are included in the RpoS and GadX iModulons while “greed” genes are those in the Translation and ppGpp iModulons.


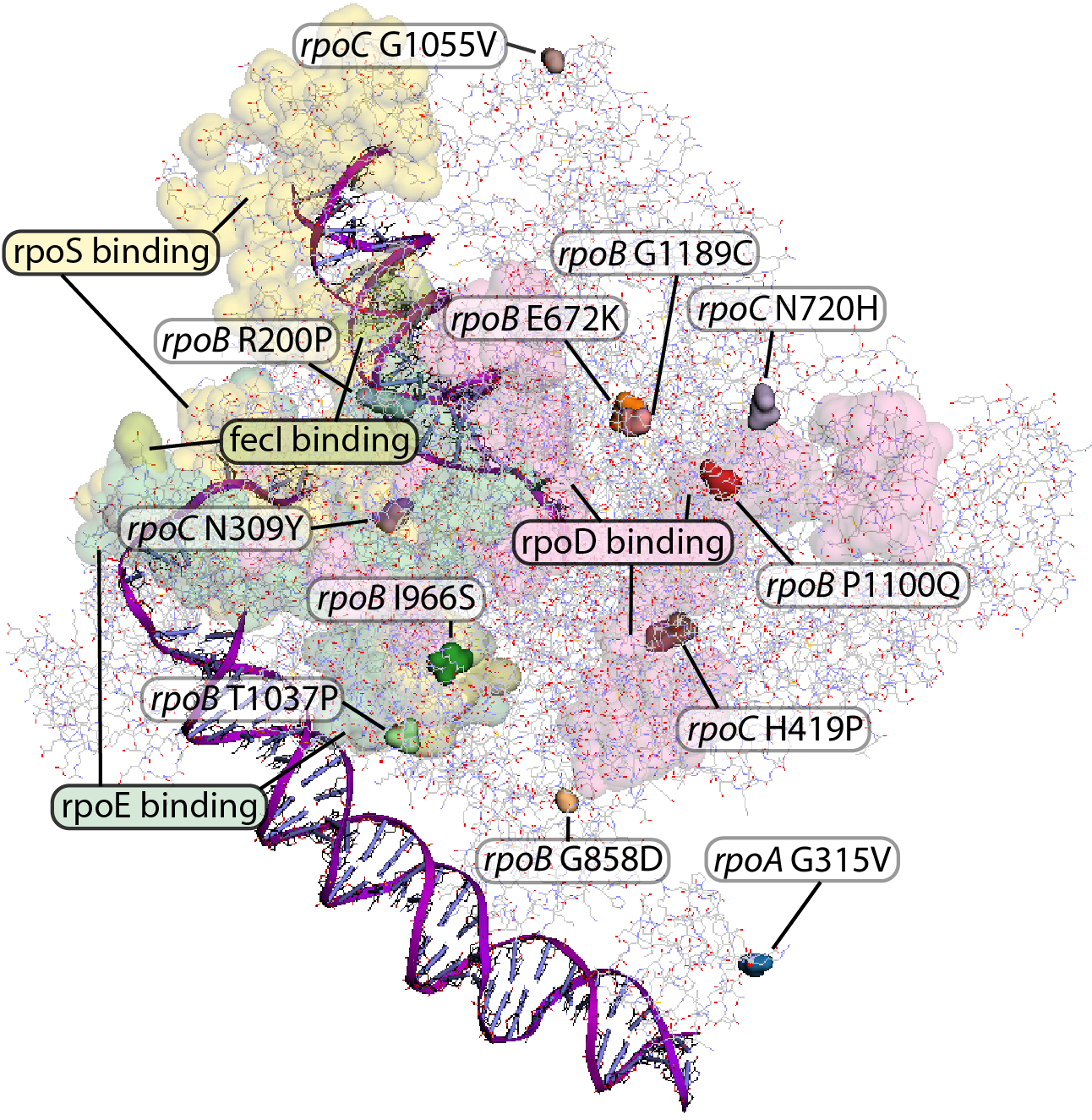


**Supplemental Figure 2 - Location of this study’s mutations and sigma factor binding sites.** The structure of RNAP (PDB 6OUL[^8^](https://paperpile.com/c/NbCbSQ/siMJ)) is visualized using PyRosetta[^30^](https://paperpile.com/c/NbCbSQ/2ydc3), showing the location of mutations used in this study and highlighting some specific RNAP regions of interest. Binding sites are inferred based on the highlighted RNAP residues being within 5 angstrom of said sigma factors in the structural files for RNAP binding with fecI (6JBQ), rpoD (6PST, 6PSR, 6PSQ, 6XLl, 6XL5, 6XL9), rpoE (6JBQ), and rpoS (5IPL, 6KJ6, 6OMF).


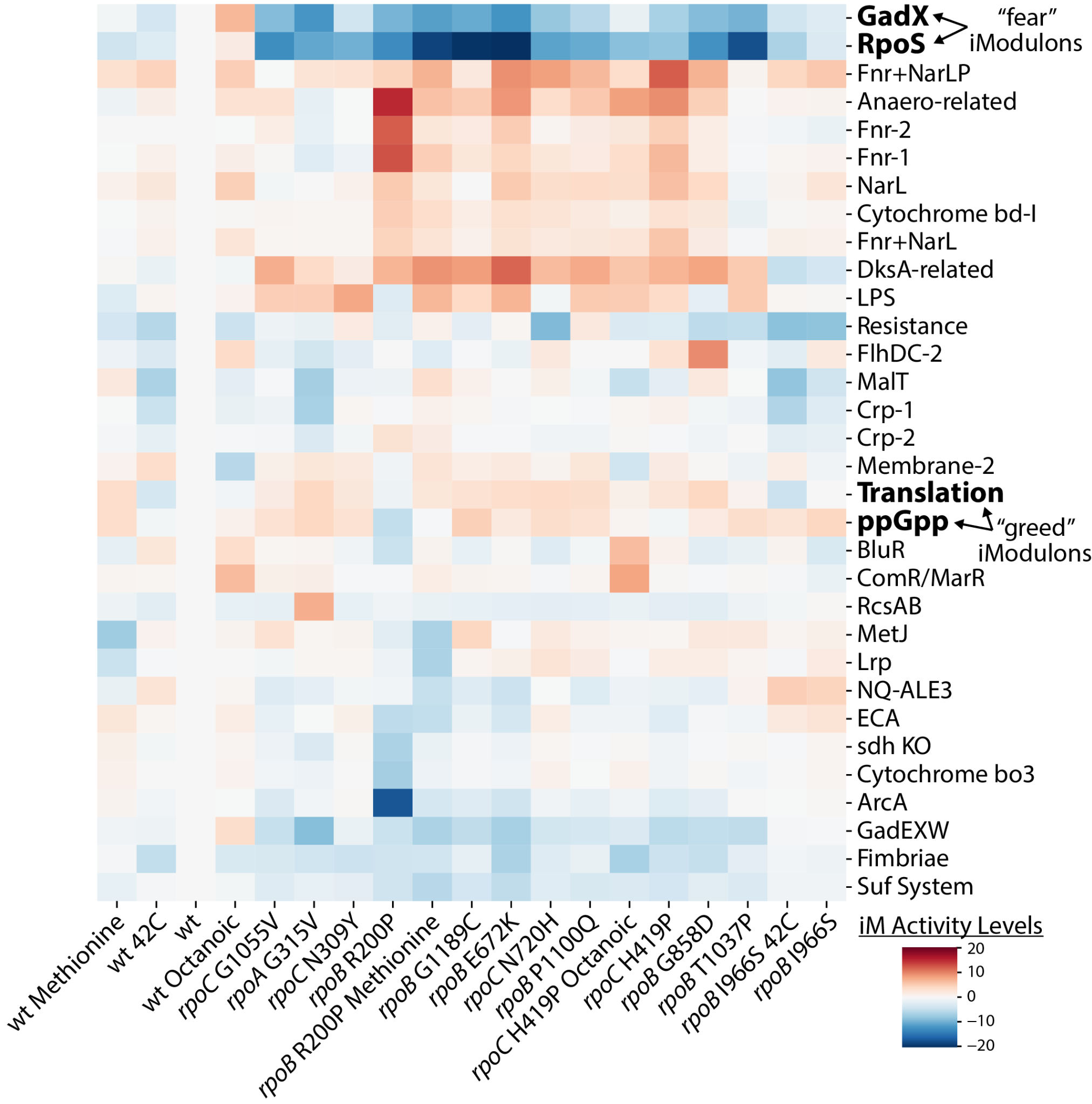


**Supplemental Figure 3 - RNAP mutations’ largest effects to iModulons.** The most differentially activated iModulons for the mutations and reference conditions are shown here. While RpoS is the strongest effect, some other iModulons are modified. The 30 highest variance iModulons for the listed samples are shown along with Crp-1 and Crp-2.


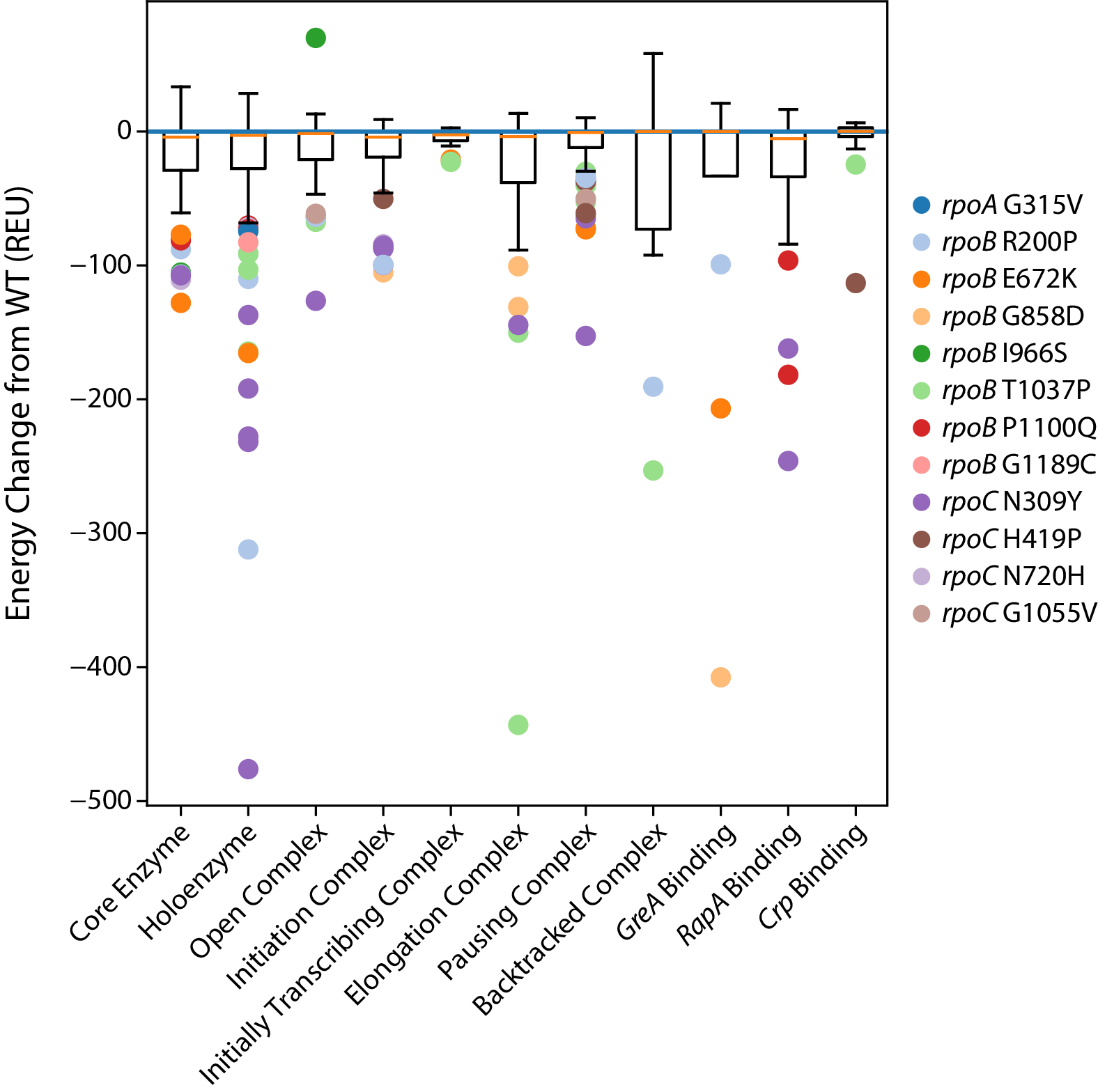


**Supplemental Figure 4 - Predicted structural impact of RNAP mutations on different forms of RNAP.** PDB structures used for each form of RNAP are listed in **Supplemental Table 3**. Nearly all mutations destabilize all of the complexes, with some mutations preferentially destabilizing certain forms. Outliers from boxplot are shown with the box representing the middle two quartiles and the whiskers stretching to 1.5 times the interquartile range.


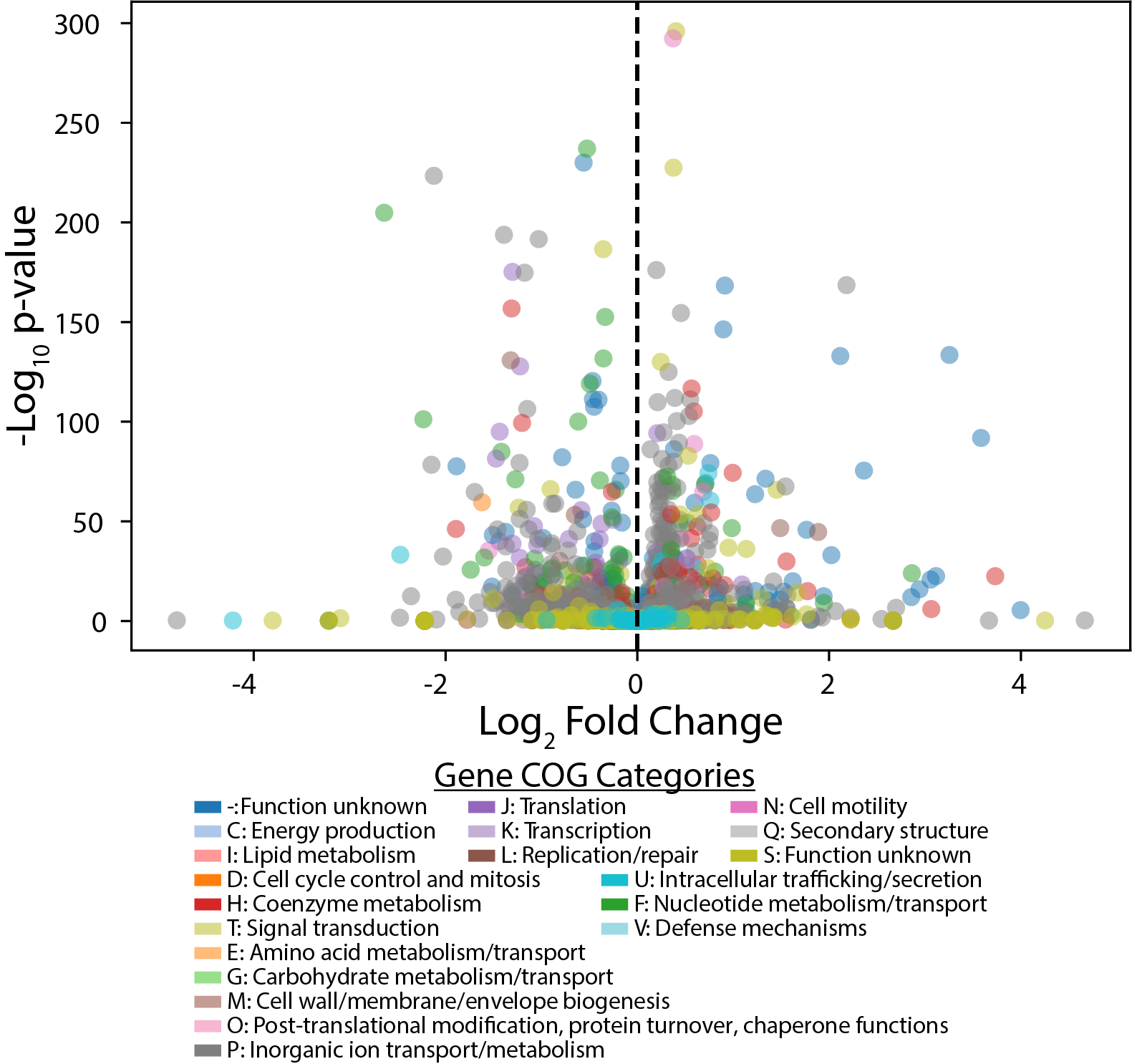


**Supplemental Figure 5 - Differential expression genes (DEG) plot between wild-type and the mutated strains colored by COG categories.** The median expression value from the mutated strains was used for the mutated strain values. The pairwise single mutant strain compared to the wild-type strain versions of this plot look similar. Interpreting these individual plots is highly difficult and doing so for all these plots together is nearly impossible, thus necessitating the use of iModulon analysis.


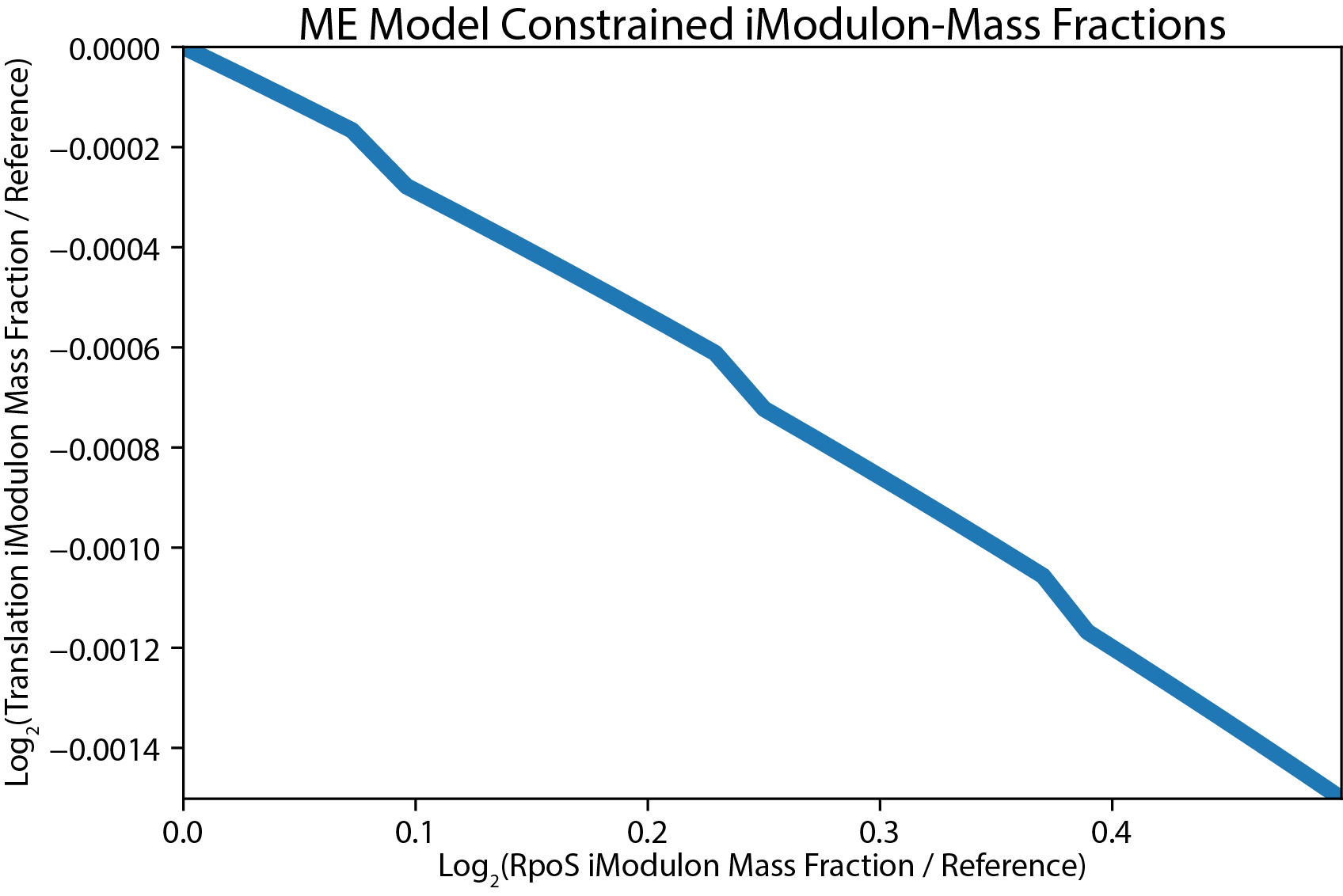


**Supplemental Figure 6 - Constrained ME-model simulation predicts fear vs. greed tradeoff.** Reactions associated with the Translation iModulon’s genes were tightly controlled in a ME model simulation, resulting in a corresponding change in the proteomic mass fraction in both the Translation iModulon and the RpoS iModulon. Growth rates increased nominally (<1%) as RpoS decreased. Note that given the large proteomic fraction allocated to the Translation iModulon compared to that of the RpoS iModulon, its fold-changes are numerically much smaller, but represent a notable proteome reallocation.


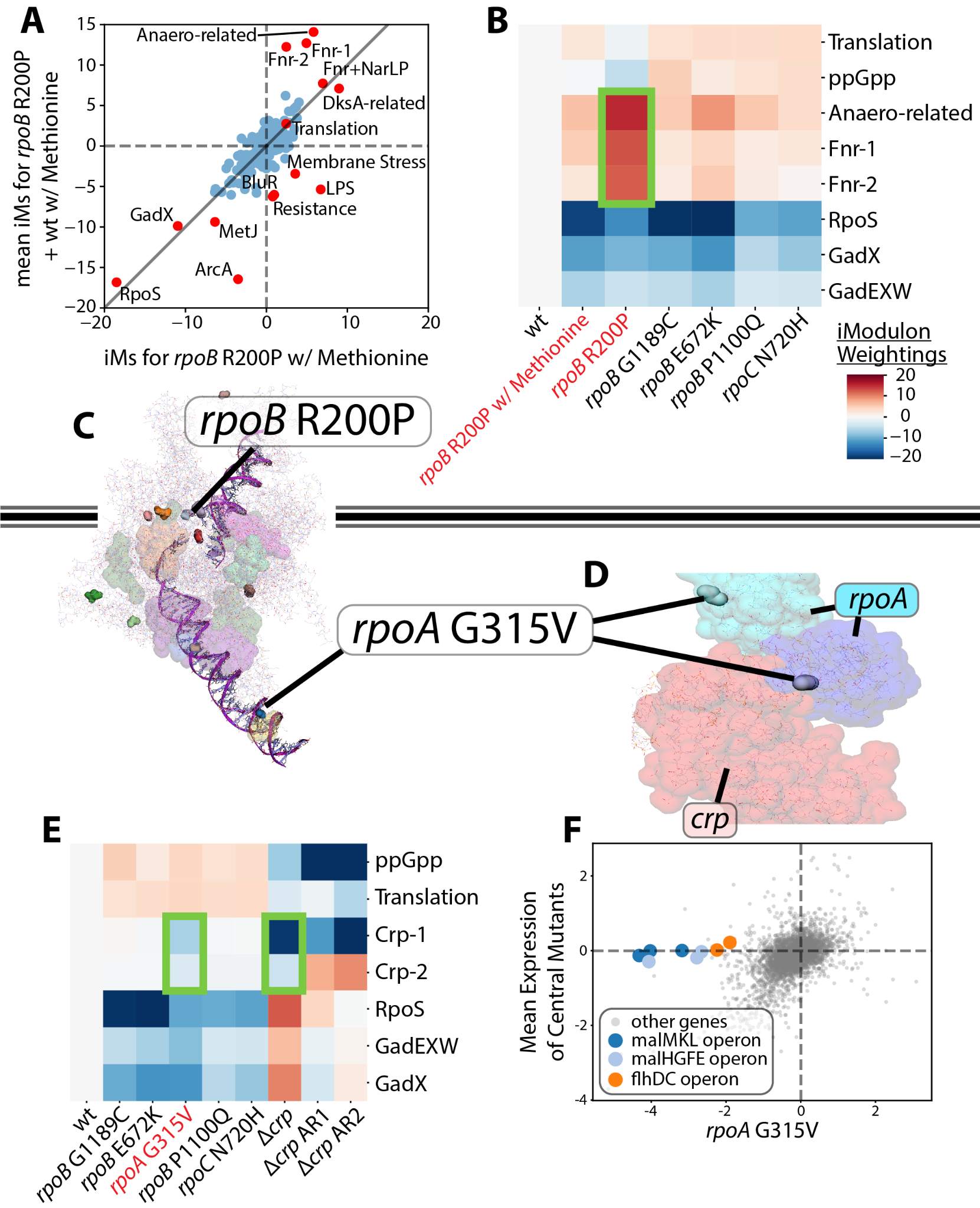


**Supplemental Figure 7 - RNAP’s ability to adjust to specific stressors. (A)** Average iModulon activities of *rpoB* R200P and wild-type supplemented with methionine compared to iModulon activities of *rpoB* R200P supplemented with methionine. This shows how the additive effects of the mutation and methionine result in a similar transcriptome to the mutation and methionine together, inferring the mutation is a specific adjustment for said condition. **(B)** *RpoB* R200P’s specific effect on Fnr and Anaero-related iModulons compared to the most common mutations. **(C)** The location of the two mutations in the whole protein (PDB 6OUL[^8^](https://paperpile.com/c/NbCbSQ/siMJ)). **(D)** Location of the *rpoA* mutation in relationship to crp (PDB 3N4M[^31^](https://paperpile.com/c/NbCbSQ/1nxUx)). **(E)** *RpoA* G315V’s effect on the iModulons, compared to the most common mutations and crp modified strains[^7^](https://paperpile.com/c/NbCbSQ/nsHRc). (F) Log tpm expression profile of *rpoA* G315V compared to the mean expression of the central mutants.


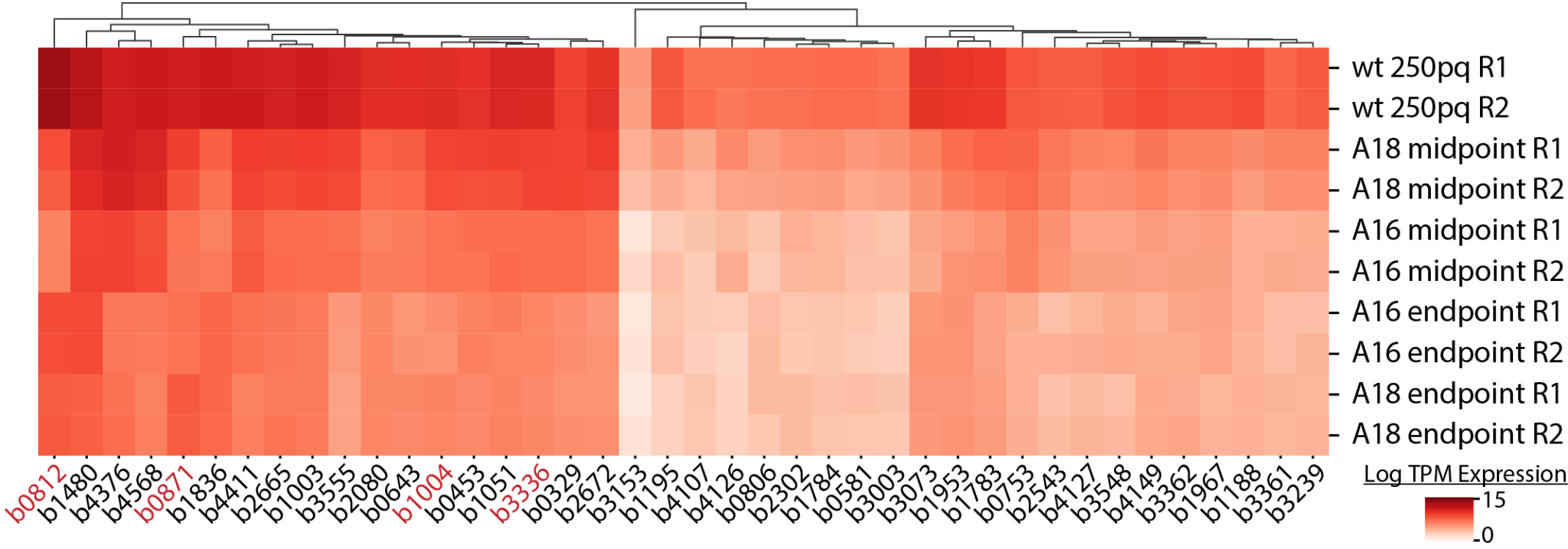


**Supplemental Figure 8 - RpoS iModulon’s genes downregulating specifically over the evolution.** As the cells evolve on 250 µM paraquat, many genes are downregulated from the RpoS iModulon to enable higher growth, but those related to oxidative stress are not (those highlighted red). Genes listed here are the top 40 most variant within the RpoS iModulon for these strains.


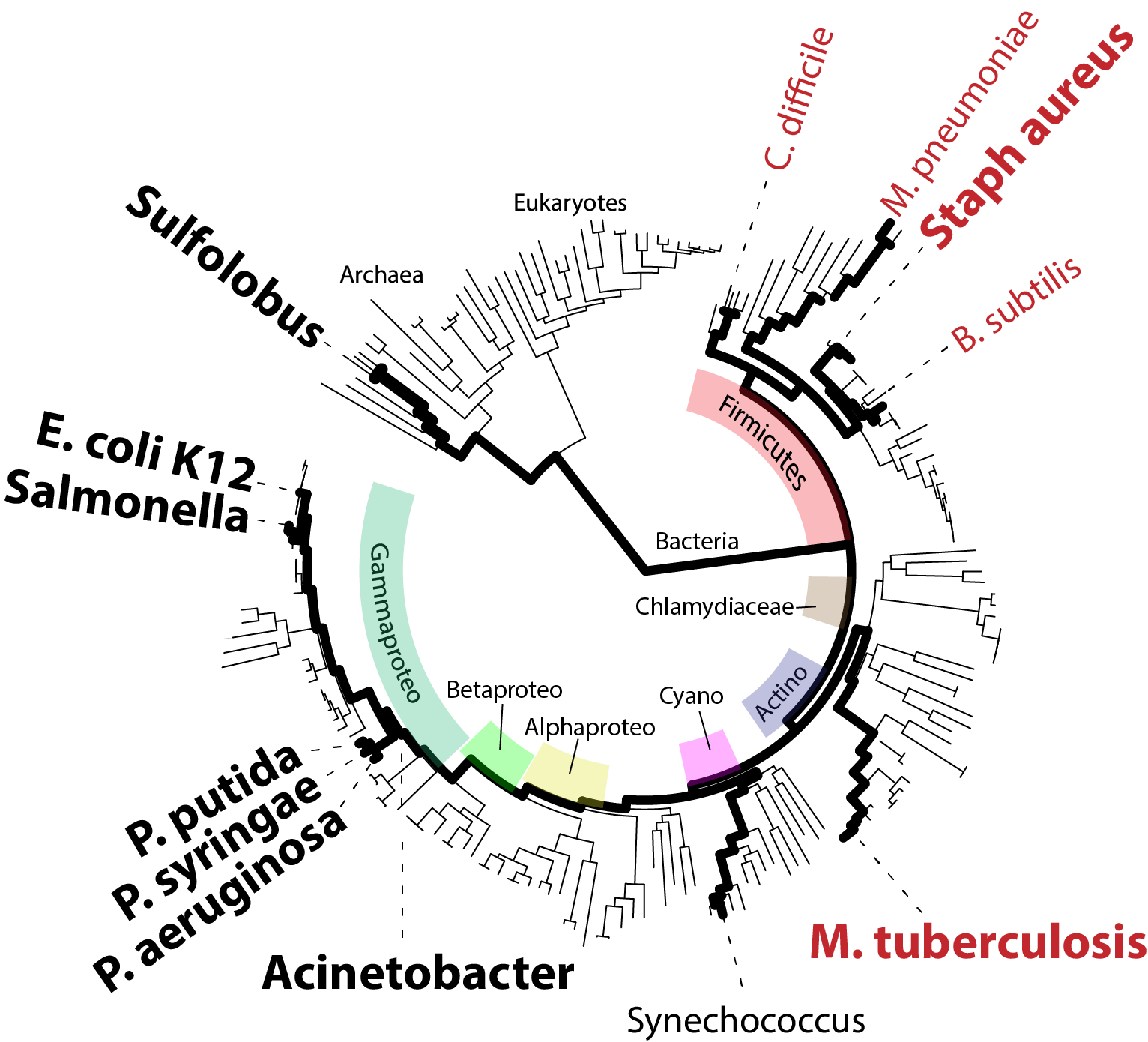


**Supplemental Figure 9 - Phylogenetic distribution of the fear vs. greed tradeoff.** All species named here were investigated for the existence of said fear vs. greed tradeoff. Red species names are gram positive, black are gram negative. The larger and bolded species names are those that are shown in **Figure 5**. Species not shown in said figure typically did not have one clear stress iModulon, making a two dimensional visualization of the fear vs. greed comparison difficult.


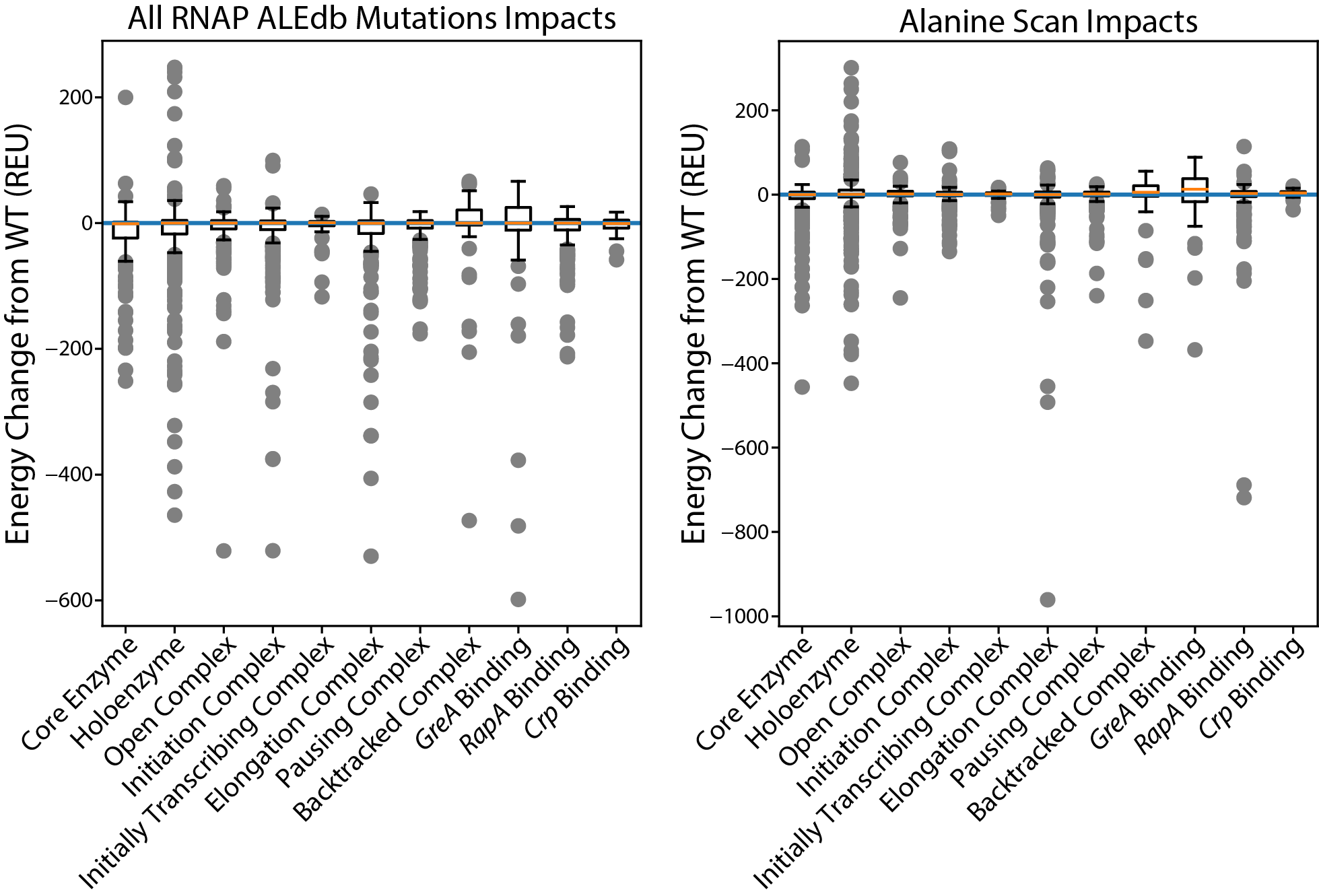


**Supplemental Figure 10 - Predicted structural impact of all ALEdb RNAP mutations and an alanine scan on different forms of RNAP.** PDB structures used for each form of RNAP are listed in **Supplemental Table 3**. Outliers from boxplot are shown with the box representing the middle two quartiles and the whiskers stretching to 1.5 times the interquartile range.


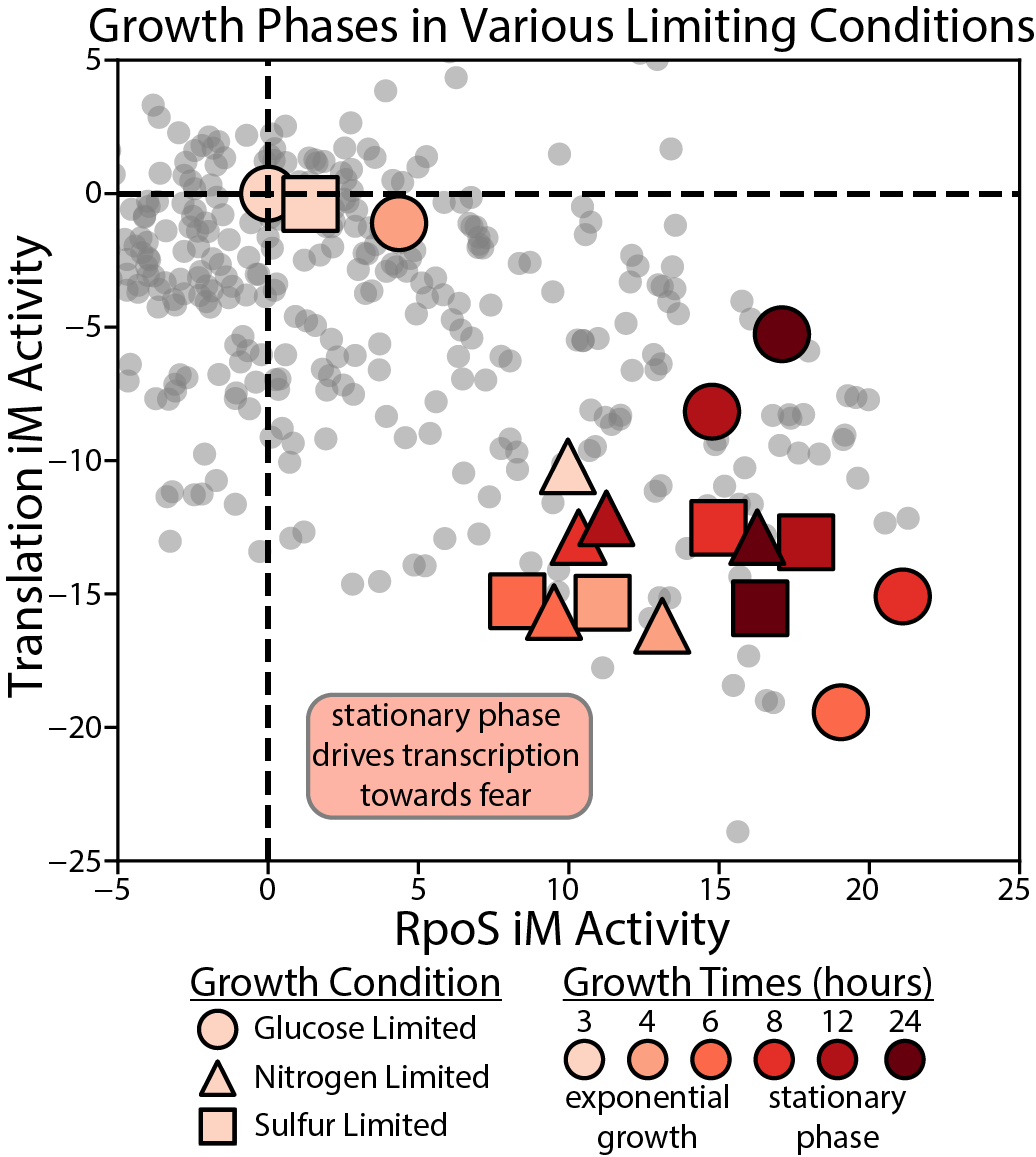


**Supplemental Figure 11 - The transition from growth to stationary phase reflected in the fear vs. greed tradeoff.** This transition comes with down regulation of the Translation iModulon and upregulation of stress iModulons. This behavior shows clearly how growth-related genes are downregulated and stress readiness increases in transition to stationary phase, the opposite change to what happens during laboratory evolution to high growth rates. Data is from NCBI GEO GSE226643.

# Bibliography

1. [Chen, Y. *et al.* Reverse engineering of fatty acid-tolerant Escherichia coli identifies design strategies for robust microbial cell factories. *Metab. Eng.* **61**, 120–130 (2020).](http://paperpile.com/b/NbCbSQ/y1ATZ)

2. [González-González, A., Hug, S. M., Rodríguez-Verdugo, A., Patel, J. S. & Gaut, B. S. Adaptive Mutations in RNA Polymerase and the Transcriptional Terminator Rho Have Similar Effects on Escherichia coli Gene Expression. *Mol. Biol. Evol.* **34**, 2839–2855 (2017).](http://paperpile.com/b/NbCbSQ/Vv9k7)

3. [Rodríguez-Verdugo, A., Tenaillon, O. & Gaut, B. S. First-Step Mutations during Adaptation Restore the Expression of Hundreds of Genes. *Mol. Biol. Evol.* **33**, 25–39 (2016).](http://paperpile.com/b/NbCbSQ/jOEDg)

4. [Tenaillon, O. *et al.* The molecular diversity of adaptive convergence. *Science* **335**, 457–461 (2012).](http://paperpile.com/b/NbCbSQ/9KGBE)

5. [Anand, A. *et al.* OxyR Is a Convergent Target for Mutations Acquired during Adaptation to Oxidative Stress-Prone Metabolic States. *Mol. Biol. Evol.* **37**, 660–667 (2020).](http://paperpile.com/b/NbCbSQ/L13aj)

6. [Houser, J. R. *et al.* Controlled Measurement and Comparative Analysis of Cellular Components in E. coli Reveals Broad Regulatory Changes in Response to Glucose Starvation. *PLoS Comput. Biol.* **11**, e1004400 (2015).](http://paperpile.com/b/NbCbSQ/eGo4y)

7. [Latif, H. *et al.* ChIP-exo interrogation of Crp, DNA, and RNAP holoenzyme interactions. *PLoS One* **13**, e0197272 (2018).](http://paperpile.com/b/NbCbSQ/nsHRc)

8. [Chen, J. *et al.* TraR allosterically regulates transcription initiation by altering RNA polymerase conformation. *Elife* **8**, (2019).](http://paperpile.com/b/NbCbSQ/siMJ)

9. [Jovanovic, M. *et al.* Activity map of the Escherichia coli RNA polymerase bridge helix. *J. Biol. Chem.* **286**, 14469–14479 (2011).](http://paperpile.com/b/NbCbSQ/npCyW)

10. [Deighan, P., Diez, C. M., Leibman, M., Hochschild, A. & Nickels, B. E. The bacteriophage lambda Q antiterminator protein contacts the beta-flap domain of RNA polymerase. *Proc. Natl. Acad. Sci. U. S. A.* **105**, 15305–15310 (2008).](http://paperpile.com/b/NbCbSQ/t0Ha8)

11. [Wang, D., Bushnell, D. A., Westover, K. D., Kaplan, C. D. & Kornberg, R. D. Structural basis of transcription: role of the trigger loop in substrate specificity and catalysis. *Cell* **127**, 941–954 (2006).](http://paperpile.com/b/NbCbSQ/7dEke)

12. [Ross, W. *et al.* ppGpp Binding to a Site at the RNAP-DksA Interface Accounts for Its Dramatic Effects on Transcription Initiation during the Stringent Response. *Molecular Cell* vol. 62 811–823 Preprint at https://doi.org/](http://paperpile.com/b/NbCbSQ/765pJ)[10.1016/j.molcel.2016.04.029](http://dx.doi.org/10.1016/j.molcel.2016.04.029) [(2016).](http://paperpile.com/b/NbCbSQ/765pJ)

13. [Tierrafría, V. H. *et al.* RegulonDB 11.0: Comprehensive high-throughput datasets on transcriptional regulation in Escherichia coli K-12. *Microbial Genomics* vol. 8 Preprint at https://doi.org/](http://paperpile.com/b/NbCbSQ/YA7HU)[10.1099/mgen.0.000833](http://dx.doi.org/10.1099/mgen.0.000833) [(2022).](http://paperpile.com/b/NbCbSQ/YA7HU)

14. [Sandberg, T. E. *et al.* Evolution of Escherichia coli to 42 °C and Subsequent Genetic Engineering Reveals Adaptive Mechanisms and Novel Mutations. *Molecular Biology and Evolution* vol. 31 2647–2662 Preprint at https://doi.org/](http://paperpile.com/b/NbCbSQ/1n3vR)[10.1093/molbev/msu209](http://dx.doi.org/10.1093/molbev/msu209) [(2014).](http://paperpile.com/b/NbCbSQ/1n3vR)

15. [Seo, S. W., Kim, D., O’Brien, E. J., Szubin, R. & Palsson, B. O. Decoding genome-wide GadEWX-transcriptional regulatory networks reveals multifaceted cellular responses to acid stress in Escherichia coli. *Nat. Commun.* **6**, 7970 (2015).](http://paperpile.com/b/NbCbSQ/wH4VL)

16. [Kim, D. *et al.* Systems assessment of transcriptional regulation on central carbon metabolism by Cra and CRP. *Nucleic Acids Res.* **46**, 2901–2917 (2018).](http://paperpile.com/b/NbCbSQ/AYnWA)

17. [Guzmán, G. I. *et al.* Enzyme promiscuity shapes evolutionary innovation and optimization. Preprint at https://doi.org/](http://paperpile.com/b/NbCbSQ/EUxlm)[10.1101/310946](http://dx.doi.org/10.1101/310946)[.](http://paperpile.com/b/NbCbSQ/EUxlm)

18. [Guzmán, G. I. *et al.* Reframing gene essentiality in terms of adaptive flexibility. *BMC Syst. Biol.* **12**, 143 (2018).](http://paperpile.com/b/NbCbSQ/u9XL1)

19. [Seo, S. W. *et al.* Deciphering Fur transcriptional regulatory network highlights its complex role beyond iron metabolism in Escherichia coli. *Nat. Commun.* **5**, 4910 (2014).](http://paperpile.com/b/NbCbSQ/fUo7u)

20. [LaCroix, R. A. *et al.* Use of Adaptive Laboratory Evolution To Discover Key Mutations Enabling Rapid Growth of Escherichia coli K-12 MG1655 on Glucose Minimal Medium. *Applied and Environmental Microbiology* vol. 81 17–30 Preprint at https://doi.org/](http://paperpile.com/b/NbCbSQ/APL8R)[10.1128/aem.02246-14](http://dx.doi.org/10.1128/aem.02246-14) [(2015).](http://paperpile.com/b/NbCbSQ/APL8R)

21. [Anand, A. *et al.* Adaptive evolution reveals a tradeoff between growth rate and oxidative stress during naphthoquinone-based aerobic respiration. *Proc. Natl. Acad. Sci. U. S. A.* **116**, 25287–25292 (2019).](http://paperpile.com/b/NbCbSQ/Utkna)

22. [Seo, S. W. *et al.* Revealing genome-scale transcriptional regulatory landscape of OmpR highlights its expanded regulatory roles under osmotic stress in Escherichia coli K-12 MG1655. *Sci. Rep.* **7**, 2181 (2017).](http://paperpile.com/b/NbCbSQ/uNVqY)

23. [Seo, S. W., Kim, D., Szubin, R. & Palsson, B. O. Genome-wide Reconstruction of OxyR and SoxRS Transcriptional Regulatory Networks under Oxidative Stress in Escherichia coli K-12 MG1655. *Cell Rep.* **12**, 1289–1299 (2015).](http://paperpile.com/b/NbCbSQ/WUg5P)

24. [Utrilla, J. *et al.* Global Rebalancing of Cellular Resources by Pleiotropic Point Mutations Illustrates a Multi-scale Mechanism of Adaptive Evolution. *Cell Syst* **2**, 260–271 (2016).](http://paperpile.com/b/NbCbSQ/f5alj)

25. [Anand, A. *et al.* Pseudogene repair driven by selection pressure applied in experimental evolution. *Nat Microbiol* **4**, 386–389 (2019).](http://paperpile.com/b/NbCbSQ/1l8TR)

26. [Rychel, K. *et al.* Lab evolution, transcriptomics, and modeling reveal mechanisms of paraquat tolerance. Preprint at https://doi.org/](http://paperpile.com/b/NbCbSQ/olcim)[10.1101/2022.12.20.521246](http://dx.doi.org/10.1101/2022.12.20.521246)[.](http://paperpile.com/b/NbCbSQ/olcim)

27. [Sandberg, T. E., Lloyd, C. J., Palsson, B. O. & Feist, A. M. Laboratory Evolution to Alternating Substrate Environments Yields Distinct Phenotypic and Genetic Adaptive Strategies. *Appl. Environ. Microbiol.* **83**, (2017).](http://paperpile.com/b/NbCbSQ/PYwrx)

28. [Sandberg, T. E., Szubin, R., Phaneuf, P. V. & Palsson, B. O. Synthetic cross-phyla gene replacement and evolutionary assimilation of major enzymes. *Nat Ecol Evol* **4**, 1402–1409 (2020).](http://paperpile.com/b/NbCbSQ/BXnu8)

29. [Gao, Y. *et al.* Systematic discovery of uncharacterized transcription factors in Escherichia coli K-12 MG1655. *Nucleic Acids Res.* **46**, 10682–10696 (2018).](http://paperpile.com/b/NbCbSQ/N4c6u)

30. [Chaudhury, S., Lyskov, S. & Gray, J. J. PyRosetta: a script-based interface for implementing molecular modeling algorithms using Rosetta. *Bioinformatics* **26**, 689–691 (2010).](http://paperpile.com/b/NbCbSQ/2ydc3)

31. [Lara-Gonzalez, S. *et al.* The RNA Polymerase α Subunit Recognizes the DNA Shape of the Upstream Promoter Element. *Biochemistry* **59**, 4523–4532 (2020).](http://paperpile.com/b/NbCbSQ/1nxUx)
